# *Melissa officinalis* extract palliates redox imbalance and inflammation associated with hyperthyroidism-induced liver damage by regulating Nrf-2/ Keap-1 gene expression in γ-irradiated rats

**DOI:** 10.1186/s12906-024-04370-z

**Published:** 2024-02-01

**Authors:** Ragaa SM Kawara, Fatma SM Moawed, Yakout Elsenosi, Hussein Abd Elmaksoud, Esraa S. A. Ahmed, Omayma AR Abo-Zaid

**Affiliations:** 1 Biochemistry and Molecular Biology Department, Faculty of Vet. Med, Benha University, Banha, Egypt; 2 Health radiation research, National Center for Radiation Research and Technology, Egyptian Atomic Energy Authority, Nasr City, 11787 Cairo Egypt; 3 Radiation Biology Research, National Center for Radiation Research and Technology, Egyptian Atomic Energy Authority, Nasr City, 11787 Cairo Egypt

**Keywords:** *Melissa officinalis*, Hyperthyroidism, Liver damage, Inflammation, Nrf2/Keap1

## Abstract

**Background:**

*Melissa officinalis* (MO) is a well-known medicinal plant species used in the treatment of several diseases; it is widely used as a vegetable, adding flavour to dishes. This study was designed to evaluate the therapeutic effect of MO Extract against hyperthyroidism induced by Eltroxin and γ-radiation.

**Methods:**

Hyperthyroidism was induced by injecting rats with Eltroxin (100 µg/kg/ day) for 14 days and exposure to γ-radiation (IR) (5 Gy single dose). The hyperthyroid rats were orally treated with MO extract (75 mg/kg/day) at the beginning of the second week of the Eltroxin injection and continued for another week. The levels of thyroid hormones, liver enzymes and proteins besides the impaired hepatic redox status and antioxidant parameters were measured using commercial kits. The hepatic gene expression of nuclear factor erythroid 2-related factor 2 (Nrf2) and its inhibitor Kelch-like ECH-associated protein-1(Keap-1) in addition to hepatic inflammatory mediators including tumor necrosis factor-α (TNF- α), Monocyte chemoattractant protein-1 (MCP-1) and fibrogenic markers such as transforming growth factor-beta1 (TGF-β1) were determined.

**Results:**

MO Extract reversed the effect of Eltroxin + IR on rats and attenuated the thyroid hormones. Moreover, it alleviated hyperthyroidism-induced hepatic damage by inhibiting the hepatic enzymes’ activities as well as enhancing the production of proteins concomitant with improving cellular redox homeostasis by attenuating the deranged redox balance and modulating the Nrf2/Keap-1 pathway. Additionally, MO Extract alleviated the inflammatory response by suppressing the TNF- α and MCP-1 and prevented hepatic fibrosis *via* Nrf2-mediated inhibition of the TGF-β1/Smad pathway.

**Conclusion:**

Accordingly, these results might strengthen the hepatoprotective effect of MO Extract in a rat model of hyperthyroidism by regulating the Nrf-2/ Keap-1 pathway.

## Introduction

Hyperthyroidism is a medical condition that arises from the excessive production and secretion of thyroid hormones. If left untreated, this condition may lead to various health complications, including weight loss, osteoporosis, liver oxidative damage, diabetes mellitus, and cardiovascular diseases, as reported by De Leo et al. [1]. The thyroid-liver interaction plays a crucial role in maintaining hormone balance in both organs and as such, liver dysfunction is a common occurrence in patients with thyroid disease. The standard treatment for hyperthyroidism involves the use of antithyroid drugs, such as propylthiouracil (PTU), carbimazole, and methimazole, as highlighted by Piantanida et al. [2]. The administration of propylthiouracil (PTU) has been shown to impede the synthesis of thyroid hormones and the conversion of thyroxine (T4) into triiodothyronine (T3) in the periphery. Nevertheless, patients who receive this medication exhibit a relatively low rate of remission (40–50%) and a high incidence of recurrence (30–80%) [3]. Furthermore, certain medications may elicit severe adverse effects, including cardiac hypertrophy, lung and liver congestion, and even the life-threatening risk of agranulocytosis. Radioactive iodine and thyroidectomy represent alternative treatments for hyperthyroidism, with a promising prognosis and low relapse rates. However, achieving a balance in the thyroid axis between hyperthyroidism and hypothyroidism can be challenging [4]. The dysregulation of thyroid hormone production and secretion may lead to an overall increase in metabolism, resulting in a high demand for the synthesis of chemical energy through a series of oxidation-reduction reactions. The hyperthyroid state is associated with an increase in oxidative stress in various tissues, and antioxidant defence is a potential therapeutic strategy. The enzymatic antioxidant system comprises three primary enzymes, namely superoxide dismutase (SOD), catalase (CAT), and glutathione peroxidase (GPx). This system can selectively neutralize the action of free radicals. SOD activity catalyzes the destruction of the superoxide anion radical to form hydrogen peroxide, while CAT and GPx can neutralize hydrogen peroxide. To eliminate peroxides, GPx requires reduced glutathione (GSH) as a cofactor, which is oxidized in the presence of free radicals [5].

Plants high in natural chemicals such as polyphenols, tocopherols, flavonoids, alkaloids, tannins, carotenoids, and terpenoids have been extensively explored in recent years due to their significant immunomodulatory, anticarcinogenic, and antioxidant capabilities. *M. officinalis* (MO) (lemon balm, Lamiaceae family) is a well-known traditional medicinal plant species used in the treatment of numerous ailments; it is also commonly eaten as a vegetable, adding flavour to recipes [6]. *M. Officinalis* is an excellent source of natural antioxidants; its leaves contain numerous phytochemicals, including polyphenolic components such as caffeic acid derivatives, imeric compounds, flavonoids, essential oil, and copious citral. Furthermore, the leaves contain vitamins E and C, which have significant action as free radical inactivators. Accordingly, *M.* officinalis has antioxidant and anti-inflammatory activities which alleviated rat cardiotoxicity induced by bleomycin [7] in addition to protecting the heart from I/R-induced damage and ameliorating collagen deposition and fibrosis [8]. It can also be used to treat neurosis, nervous excitability and anxiety- and depression in mice exposed to restraint stress [9]. MO is also regarded to be useful for people with Alzheimer’s disease and has therapeutic potential in mood and cognitive performance regulation [10]. Furthermore, it was found that MO ameliorated hepatic and renal dysfunction in rats intoxicated with lead [11], liver fibrosis in the non-alcoholic fatty liver disease model [12] as well as its chemo-preventive effect against HCC in rats [13]. Although multiple papers on the role of MO in the treatment of various disorders have been published, there is no available information on the effect of MO extract on the hyperthyroidism state [14].

The nuclear factor erythroid 2-related factor (Nrf2) is a redox-sensitive transcription factor that binds to the negative regulator Kelch-like ECH-associated protein 1 (Keap1); however, when oxidative stress occurs, it dissociates from Keap1 and translocates to the nucleus. Nuclear Nrf2 acts as an activator of antioxidant response elements to regulate protective genes, resulting in increased cell survival. As a result, Nrf2 has emerged as a popular research target because dysregulation of Nrf2 may give a viable therapeutic target as well as a rational explanation for the link between oxidant stress and hyperthyroidism. Furthermore, heme oxygenase (HO-1), a downstream Nrf2 target protein, is an inducible isoform of heme oxygenase that protects hepatocytes from liver injury [15]. As a result, Nrf2 and HO-1 have been identified as potential targets for preventing and treating liver injury and illness. Accordingly, MO Extract was used in this investigation to assess its preventive properties against induced hyperthyroidism in a rat model as well as its regulatory effects on deranged redox balance and inflammation as well as the Nrf2/Keap-1 pathway. The anti-hyperthyroidism action of this extract was discovered through thyroid hormone testing. We also investigated the impact of hyperthyroidism induction on liver functioning.

## Materials and methods

### Chemicals and drugs

Eltroxin (levothyroxine sodium; 100 µg; 100 tablets) is commercially available and was purchased from GlaxoSmithKline Co. (New Cairo City, Cairo, Egypt). All other chemicals were of the highest analytical grade available and obtained from Sigma Aldrich Chemical Co. (St. Louis, USA).

### Plant materials

*Melissa officinalis* an aromatic herb of the mint family (Lamiaceae), was obtained from Abd El-Rahman Harraz (Bab El-Khalk Zone, Cairo, Egypt). A plant was identified at Al-Azahr University, Faculty of Science, (Boys) by Abdel-Aziz [16], who and others reported that *M. officinalis* contains high amounts of flavonoids, rosmaric acid, gallic acid, and phenolic contents [17].

### Methods

#### *M. officinalis* extraction

For the extraction process, the herbal leaves were air-dried, ground, and immersed in ethanol (1: 10 w/v) for 3 days while being continuously shaken. Following filtration, the solvent was expelled using a rotary evaporator at low pressure until dryness was attained. The yield percentage was then determined as 1 g (extract)/100 g. (crude powdered herb). The *M. officinalis* ethanolic extract (MO Extract) was kept at -20 °C until use according to the method described by Mannaa et al. [14].

#### Experimental animals

Twenty-four Wister male albino rats weighing 120–150 g at the age of three months were obtained from the Nile Company’s breeding unit in Egypt. Rats were housed under standard conditions of humidity (50 ± 5%) and a 12:12-h light-dark cycle for one week before starting the experiment as an acclimatization period. Animals were freely fed on starter poultry pellets and water *ad libitum.*

### Ethics approval

The handling and treatment of the experimental animals were consistent with the guidelines of the National Research Center Ethics Committee published by the U.S. National Health Institutes (NIH publication No. 85 − 23, 1996). This study was approved by the Institutional Animal Care and Use Committee Research Ethic Board of Benha University, Faculty of Veterinary Medicine (BUFVTM 05-04- 022).

### Irradiation process

At the National Centre for Radiation Research and Technology (NCRRT, Cairo, Egypt), whole-body gamma irradiation was performed using Canadian gamma cell-40 (137Cesium) at a dose rate of 0.333 Gy•min^-1^ for a total dose of 5 Gy in a single dose [18].

### Induction of hyperthyroidism

Hyperthyroidism was induced by daily subcutaneous injection of Eltroxin (Elt.) (100 µg/kg) [19] for 14 days according to the method of Carageorgiou et al. [20]. After the last dose of Eltroxin injection rats were exposed to a single whole-body gamma radiation at a dose of 5 Gy.

### Experimental groups

After the acclimatization period, rats were divided into four groups (*n* = 6) as follows: Control group: included normal rats that were subcutaneously (s.c.) given 0.5 ml physiological saline. MO extract group: included the rats that were orally treated with MO Extract (75 mg/kg/day) for two weeks (14 days) [16]. Eltroxin + IR group: included untreated Eltroxin + IR-induced hyperthyroid rats. Eltroxin + IR + MO extract group: included the hyperthyroid rats that were orally treated with MO extract (75 mg/kg/day) at the beginning of the second week of the Eltroxin injection and continued for another week.

At the end of the experimental period, the animals were fasted overnight and sacrificed under anaesthesia using urethane. Blood samples were collected from each rat and were centrifuged at 3000 rpm for 15 min to obtain serum. After that, liver tissues were dissected out and washed with saline, dried on filter paper, and then divided into two parts. The first part from each liver was stored at − 80 °C for the biochemical determinations after homogenization in an ice-cold phosphate buffer solution (pH 7.4). Moreover, the other part of the liver was immediately preserved in a 10% buffered formalin-saline solution for a later histopathological examination.

### Biochemical determinations

Serum thyroid stimulating hormone (TSH; No. MBS701641), total triiodothyronine (T3; No MBS261285), and total thyroxine (T4; No MBS704309) levels were determined by enzyme-linked immunosorbent assay (ELISA) using rat ELISA kits purchased from My BioSource Co. (San Diego, California, USA).

Using a commercial kit from Bio-diagnostic Company (Cairo, Egypt) the malondialdehyde content as the end product of lipid peroxidation (MDA; No. MD 25 29), the level of reduced glutathione (GSH; No. GR 25 11), the activity of glutathione peroxidase (GPx; No. GP 25 24), catalase (CAT; No. CA 25 17), superoxide dismutase (SOD; No. SD 25 21) and nitric oxide level (NO; No. NO 25 33) were measured calorimetrically in the liver homogenates.

### Liver function biomarkers in serum

Activities of alanine aminotransferase (ALT, EC 2.6.1.2; Catalog Numbers: 264 001, 264 002) and aspartate aminotransferase (AST, EC 2.6.1.1; Catalog Numbers: 260 001, 260 002) were measured spectrophotometrically using commercial kits purchased from Spectrum Diagnostic Company Cairo, Egypt. Moreover, the levels of total protein (TP), albumin (Alb) and globulin were measured in serum calorimetrically according to previous methods [21, 22] respectively.

### Quantitative real-time polymerase chain reaction (RT-qPCR)

Total RNA Purification Kit (Thermo Scientific, Ferments, #K0731) was used for the extraction of the pure RNA from the hepatic tissues. The complementary DNAs were synthesized using a Revert Aid First Strand cDNA Synthesis Kit and reverse transcription kits (Thermo Scientific, Ferments, # EP0451) following the manufacturer’s instructions. Using RT-PCR with SYBR Green the quantitative expression of the studied genes was measured. The isolated cDNA was amplified using 2× Maxima SYBR Green/ROX qPCR Master Mix and gene-specific primers, as directed by the manufacturer (Thermo Scientific, USA, # K0221). The primer sequence is shown in Table 1. Under adjusted conditions (95 °C for 10 min, 60 °C for 30 s and 72 °C for 30 s) the thermal cycler was used for 40 cycles. The relative expression of mRNA of each gene in each sample was calculated using the 2^−ΔΔCT^ method [23]. β- actin was used as an internal control gene.

**Table 1:** Sequences for primers

Gene	Forward primer (^/^5 ------ ^/^3)	Reverse primer (^/^5 ------ ^/^3)
TNF-α	GCATGATCCGCGACGTGGAA	AGATCCATGCCGTTGGCCAG
TGFβ1	AAGAAGTCACCCGCGTGCTA	TGTGTGATGTCTTTGGTTTTGTCA
MCP-1	TCGCTTCTGACACCATGCA	TGCTACAGGCAGCAAATGTGA
NrF2	CACATCCAGACAGACACCAGT	CTACAAATGGGAATGTCTCTGC
Keap-1	*GGACGGCAACACTGATTC*	*TCGTCTCGATCTGGCTCATA*
SDF1	AGAGCCAACGTCAAGCATCT	GGGCAGCCTTTCTCTTCTTC
α-SMA	GAGGCACCACTGAACCCTAA	CATCTCCAGAGTCCAGCACA
***B-actin***	AAGTCCCTCACCCTCCCAAAAG	AAGCAATGCTGTCACCTTCCC

TNF-α: tumor necrosis factor-α, TGF-β1: transforming growth factor-beta1, MCP-1 (Monocyte chemoattractant protein-1, Nrf2: nuclear factor erythroid 2-related factor, Keap-1: Kelch-like ECH-associated protein-1, SDF-1: Stromal cell-derived factor-1, α-SMA: alpha-smooth muscle actin

### Histopathological examination

Liver tissue samples were collected and fixed in a 10% neutral buffered formalin solution for histopathology. Tissue specimens were processed as follows: dehydrated in an ascending concentration of ethanol, cleared in xylene, embedded in paraffin wax, and sectioned at a 5-micron thickness [24]. Prepared slide sections were stained with hematoxylin and eosin and examined by a light digital microscope (Olympus XC30, Tokyo, Japan). The frequency and severity of lesions in the liver were assessed semi-quantitatively as previously reported by Plaa et al. [25] using a scale where, grade 0: No apparent injury, grade I: Swelling of hepatocytes, grade II: Ballooning of hepatocytes, grade III: Lipid droplets in hepatocytes and grade IV: Necrosis of hepatocytes.

### Statistical analysis

The results were statistically analyzed by one-way ANOVA, followed by the Bonferroni post hoc test to assess the differences between groups at a *p* < 0.05 using the SPSS software package (SPSS 20, SPSS Inc, USA). The data are expressed as mean ± SD (*n* = 6/group). Moreover, the charts were graphed via GraphPad Prism 8 (GraphPad, CA, USA).

## Results

### Effects of MO Extract on the thyroidal -hormones status in hyperthyroidism rats

**Fig. 1:**
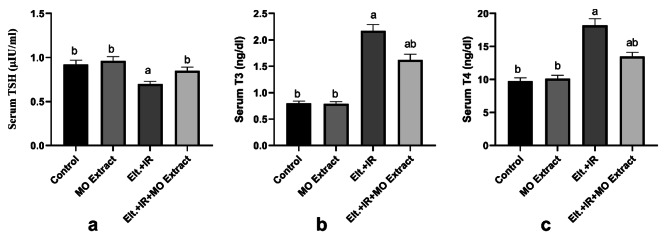
Effects of MO Extract on the serum levels of thyroid hormones. (**a**) Thyroid-stimulating hormone (TSH), (**b**) triiodothyronine (T3), and (**c**) tetraiodothyronine (T4). *N* = 6 for each group. Data are presented as mean ± SD. a, b denotes significant change at *p* ≤ 0.05 versus control and Eltroxin + IR groups, respectively

The effects of MO Extract treatment for 14 days on the blood levels of the thyroid hormones (TSH, T4, and T3) were evaluated. A notable decrease in the TSH levels was observed in the hyperthyroidism group. Moreover, the levels of T3 and T4 were significantly increased in the hyperthyroidism group relative to the control group. On the contrary, supplementation with MO Extract reversed the effect of Eltroxin + IR on the thyroid gland by elevating the level of the TSH along with lowering those of both T3 and T4 (Fig. 1) indicating the modulatory effect of the MO Extract on thyroid dysfunction and thyroid hormones status.

### Effects of MO Extract on liver function in hyperthyroidism rats

**Fig. 2:**
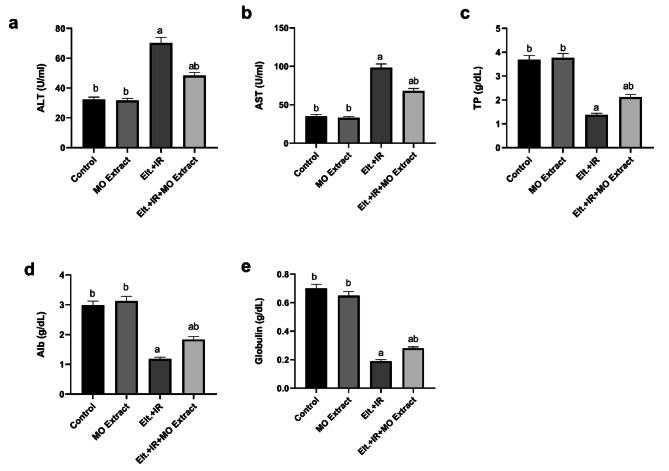
Effects of MO Extract on liver function biomarkers. (**a**) alanine transaminase (ALT), (**b**) aspartate transaminase (AST), (**c**) total protein (TP), (**d**) albumin (Alb), (**e**) globulin (Glob). *N* = 6 for each group. Data are presented as mean ± SD. a, b denotes significant change at *p* ≤ 0.05 versus control and Elt.+ IR groups, respectively

Regarding the functional relation between the thyroid gland and the liver, hyperthyroidism triggers hepatic dysfunction. Herein, as a consequence of hyperthyroidism a pronounced increment in the activities of both ALT and AST in the Elt. + IR group compared to the control. Moreover, these hyperthyroid rats displayed a remarkable decline in the levels of TP, Albumin and globulin relative to their normal counterparts (Fig. 2). Conversely, treatment with MO Extract effectively ameliorated the hepatic function via abolishing the activities of the hepatic enzymes (ALT and AST) concomitant with enhancing the hepatic production of the proteins (TP, Alb, and Glob.) suggesting the hepatoprotective potential of the MO Extract.

**Fig. 3:**
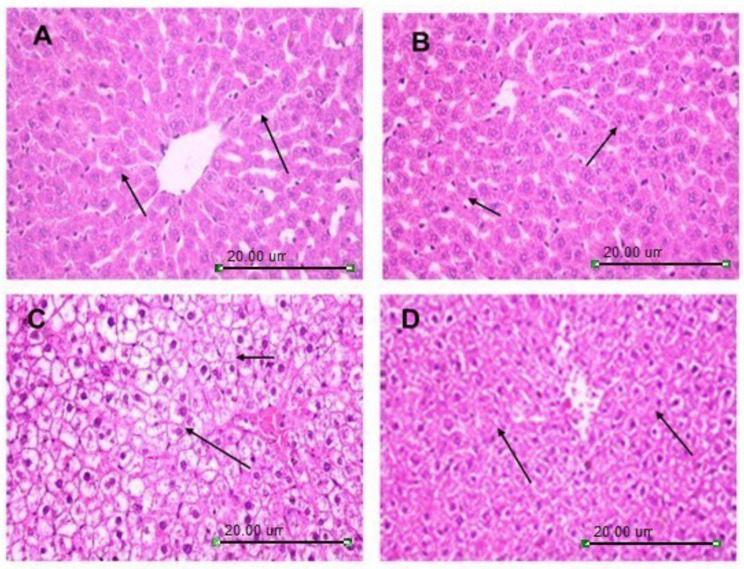
Photomicrograph of hepatic tissue section showing the normal histological architecture of the hepatic section consisting of strands of polygonal cells with prominent round nuclei, and eosinophilic cytoplasm (arrow) of both the control (**A**) and MO Extract group (**B**). Elt.+IR group (**C**): damaged structural integrity of the liver hepatocytes and intracellular fat droplets and infiltration of inflammatory cells. Elt. + IR + MO Extract group (**D**): alleviated hepatocyte degeneration concomitantly with improving the hepatic sinusoids (arrow)

Additionally, the histopathological examination of the liver tissues section of hyperthyroid rats displayed damaged structural integrity of the liver represented by ballooning degeneration of hepatocytes, coagulative necrosis of the peripheral hepatocytes, narrow sinusoidal space subsequent to Kupffer cells hyperplasia. Besides, cytoplasmic vacuolar degeneration, intracellular fat droplets and infiltration of inflammatory cells (Grade IV) (Fig. 3C) compared to the normal histological architecture of the hepatic section of both the control and MO Extract group. Both groups showed polygonal hepatic cells, hepatic lobules, hepatic cords with prominent central hepatic vein and sinusoids lined by a discontinuous layer of fenestrated endothelial cells with a fine arrangement of Kupffer cells (Grade 0) (Fig. 3A&B). Alternatively, treating the hyperthyroid rats (Elt. IR) with MO Extract alleviated the related changes by reducing the ballooning degeneration of hepatocytes, hyperplasia of Kupffer cells, foamy cytoplasm and pyknotic nuclei concomitantly with improving the hepatic sinusoids (Grade II) (Fig. 3D).

### Effect of MO Extract on the impaired hepatic redox status

**Fig. 4:**
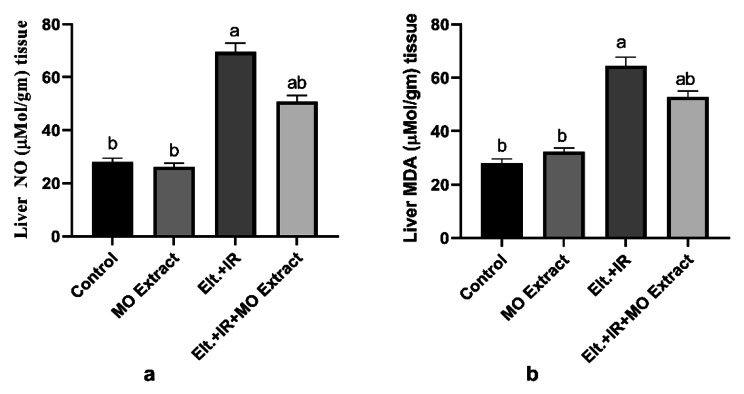
Effects of MO Extract on hepatic oxidative stress markers. (**a**) nitric oxide (NO), (**b**) lipid peroxidation (MDA). *N* = 6 for each group. Data are presented as mean ± SD. a, b denotes significant change at *p* ≤ 0.05 versus control and Elt.+IR groups, respectively

Depending on the role of the thyroid hormones in regulating metabolic processes, it was reported that hyperthyroidism is associated with the generation of the reactive oxygen species (ROS) consequently oxidative damage to body organs and molecules accompanied with deterioration of the antioxidant defence system and final impairment of the redox status. The current results demonstrated a dramatic increase in the levels of lipid peroxidation (MDA) as well as nitric oxide (NO) levels in the hepatic tissues of Elt.+ IR-induced hyperthyroidism rats compared to the control rats indicating oxidative damage to hepatic tissues as shown in Fig. 4. In contrast, upon treatment with MO Extract a significant reduction in the levels of these oxidative stress markers (MDA and NO).

### Effect of MO Extract on the antioxidant system

**Fig. 5:**
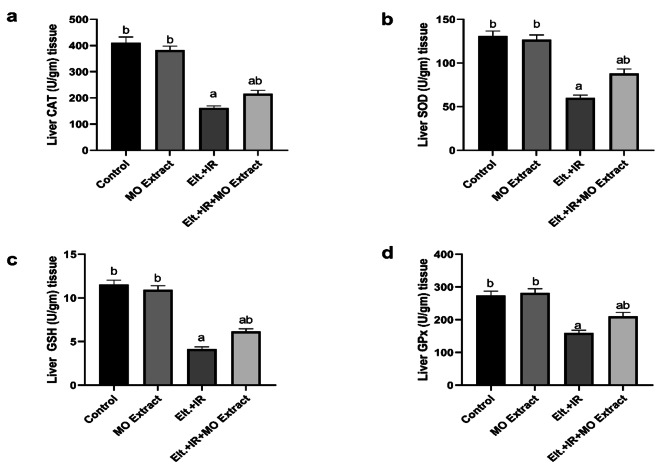
Effects of MO Extract on antioxidant markers. Activities of (**a**) catalase (CAT), (**b**) liver superoxide dismutase (SOD), (**c**) intracellular glutathione (GSH), and (**d**) glutathione peroxidase (GPx). *N* = 6 for each group. Data are presented as mean ± SD. a, b denotes significant change at *p* ≤ 0.05 versus control and Elt.+ IR groups, respectively

As shown in Fig. 5, a considerable depletion in the levels of GSH as well as the activities of the antioxidant enzymes (CAT, SOD and GPx) in the hepatic tissues of the hyperthyroid rats relative to the control rats. Meanwhile, hyperthyroid rats treated with MO Extract exhibited a pronounced amelioration in the antioxidant system by mitigating the effect of Elt.+ IR. These results exhibited the antioxidant effect of MO Extract *via* retrieving the impaired redox status.

### Effects of MO Extract on hepatic Nrf2/ Keap-1 expression

**Fig. 6:**
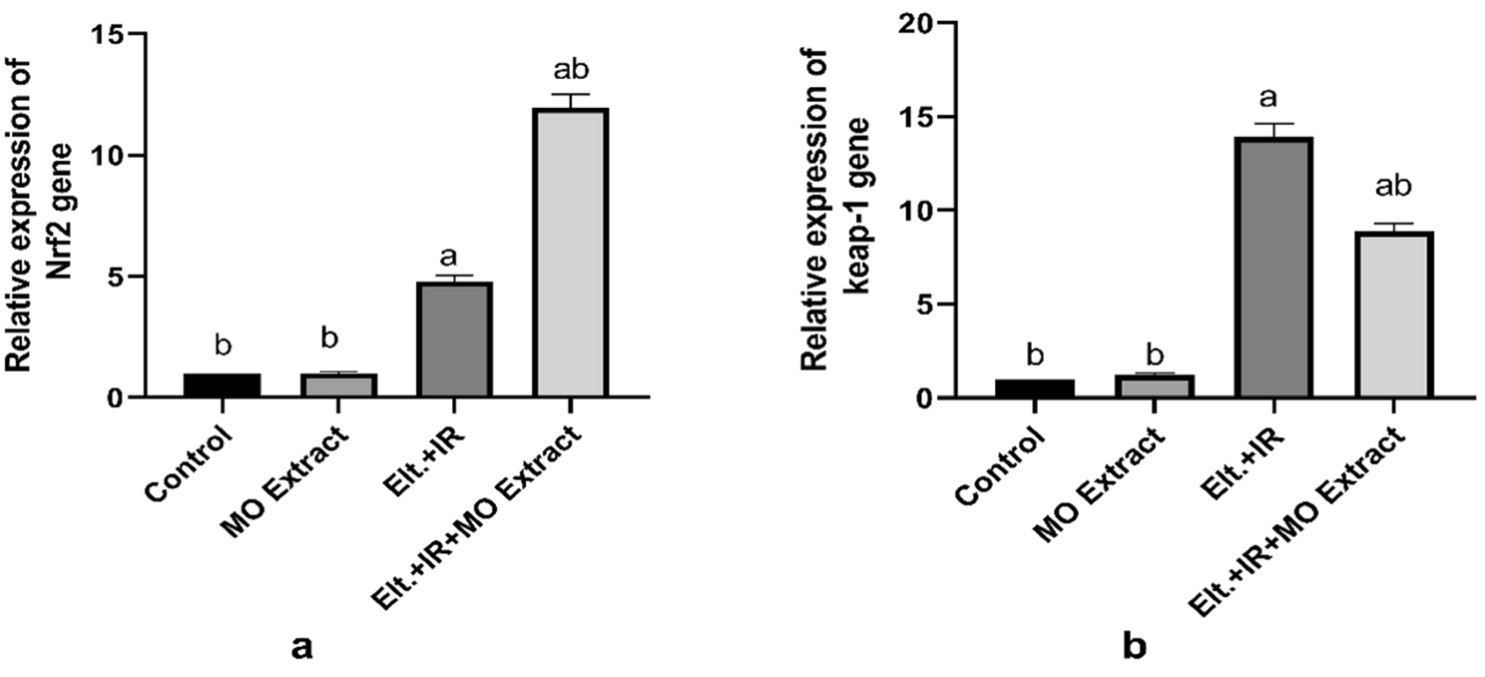
Effects of MO Extract on hepatic Nrf2/ Keap-1 expression. Expression of Nrf2 gene (**a**) and Keap-1 gene (**b**). *N* = 6 for each group. Data are presented as mean ± SD. a, b denotes significant change at *p* ≤ 0.05 versus control and Eltroxin + IR groups, respectively

Regarding the above results and consequent to the impaired redox status in the Elt.+ IR-induced hyperthyroidism, the current results revealed a moderate increase in the mRNA expression of the Nrf2 gene coupled with pronounced elevation in the expression of the Keap-1 gene compared to the control rats. conversely, treating the hyperthyroid rats with MO Extract significantly upregulated the gene expression of the Nrf2 together with suppressing that of the Keap-1 gene in the hepatic tissues (Fig. 6).

### Effects of MO Extract on hepatic inflammatory markers

**Fig. 7:**
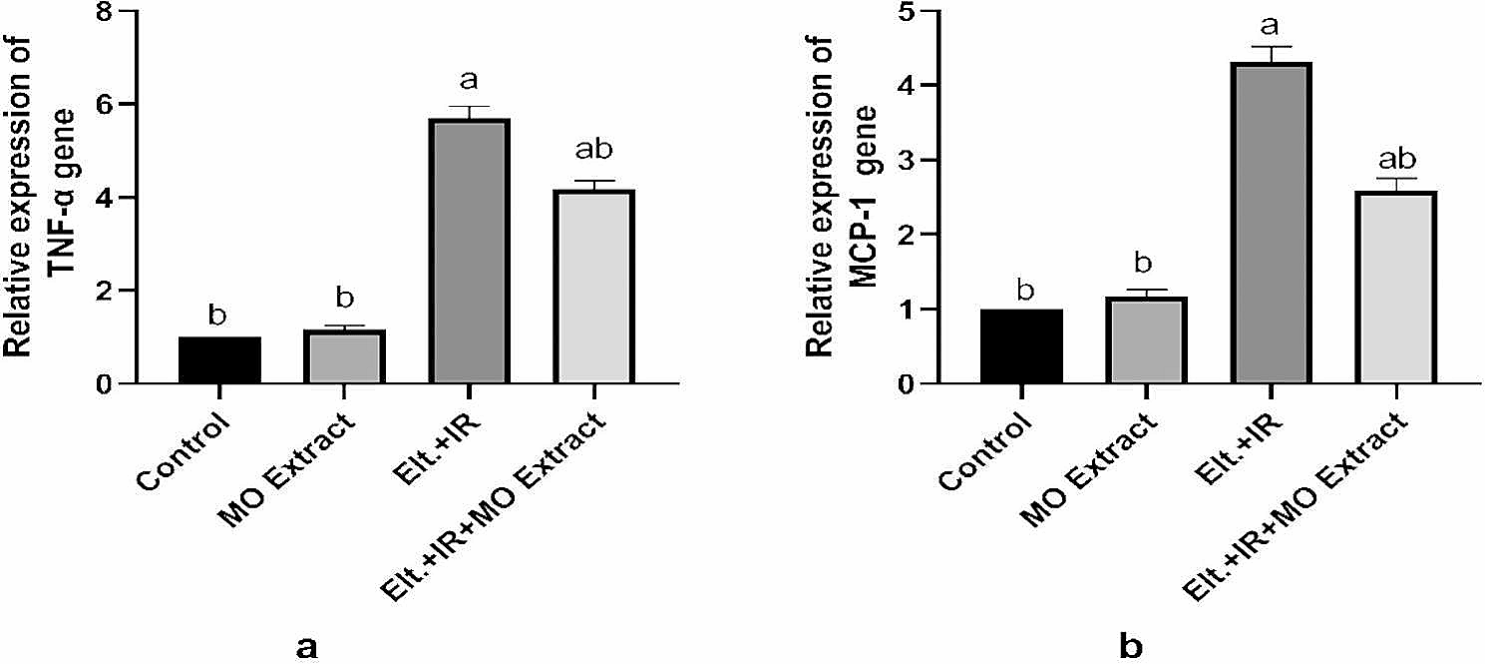
Effects of MO Extract on hepatic inflammatory markers. Gene expression of TNF-α (**a**) and MCP-1 (**b**). *N* = 6 for each group. Data are presented as mean ± SD. a, b denotes significant change at *p* ≤ 0.05 versus control and Elt.+ IR groups, respectively

The oxidative stress and production of ROS associated with hyperthyroidism trigger hepatic injury which mediates an inflammatory response in the liver. This inflammatory response is initiated with the activation of the hepatic macrophages which in turn boosts the release of the proinflammatory mediators (cytokines and chemokines). Considering this, the obtained results in Fig. 7 showed a remarkable upgrade in the gene expression of the pro-inflammatory markers (TNF-α and MCP-1) in the hepatic tissues of the hyperthyroid rats relative to control rats. Controversially, the hyperthyroid rats treated with MO Extract displayed significant blunted expression of the pro-inflammatory mediators indicating the anti-inflammatory role of MO Extract against the hyperthyroidism derived-hepatic inflammation.

#### Effect of MO Extract on hepatic fibrosis

**Fig. 8:**
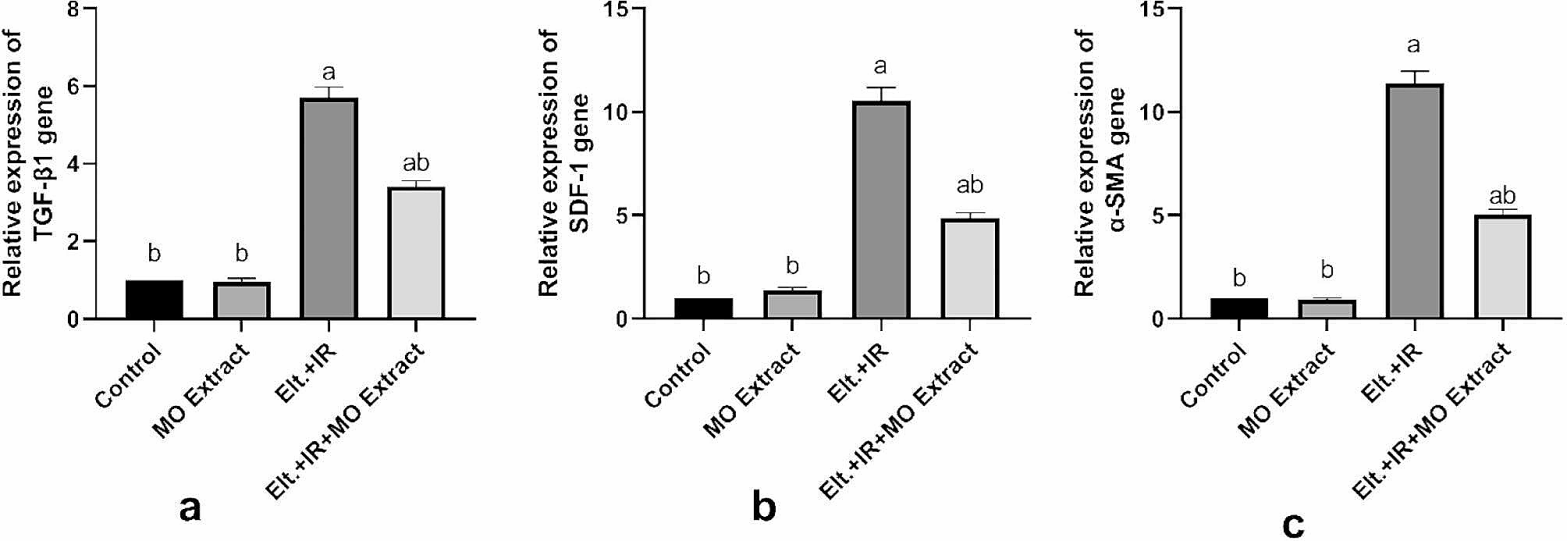
Effects of MO Extract on hepatic fibrogenic mediators. Gene expression of TGF-β1 (**a**), SDF-1 (**b**) and α-SMA (**c**). *N* = 6 for each group. Data are presented as mean ± SD. a, b denotes significant change at *p* ≤ 0.05 versus control and Elt.+IR groups, respectively

Furthermore, the elevated pro-inflammatory markers resulted in the activation and proliferation of the hepatic stellate cells (HSCs) promoting hepatic fibrosis through their released fibrogenic mediators. Accordingly, a pronounced upregulation in the mRNA expression of the pro and fibrogenic mediators including TGF-β1, SDF-1 and α-SMA was shown in the hepatic tissues of the hyperthyroid rats versus the control rats. Meanwhile, supplementation with MO Extract attenuated the hepatic fibrosis by suppressing the expression of the fibrogenic mediators (Fig. 8).

## Discussion

Aside from its low toxicity and lack of side effects, as well as the short duration of treatment [26], the natural medicinal plant M. officinalis, which has a wide range of beneficial biological and health properties, has been used effectively for the treatment of various diseases such as cardiovascular, neurological, and psychological in addition to various experimental studies [27]. Accordingly, our hypothesis was to evaluate whether MO Extract could alleviate the hyperthyroidism-induced hepatic damage by attenuation of the deranged redox balance and inflammation as well as modulation of the Nrf2/Keap-1 pathway.

Herein, the daily injection of Elt. (L-thyroxine) to rats along with exposure to a single dose of whole-body γ- radiation resulted in increased levels of both T3 and T4 accompanied by a decrease in the TSH levels in the circulating blood. This uncontrolled excessive release of thyroid hormones resulting from a hyperactive or dysfunctional gland is known as hyperthyroidism or thyrotoxicosis [28, 29] which may be attributed to the augmented effect of both Elt. and γ- radiation. Kim et al. [30] and Mullur et al. [31] indicated that L-thyroxine boosted thyroid gland activity via diminishing the oxidative iodination within the gland thus perturbing the production and release of the thyroid hormones. Moreover, it was found that thyroid damage derived from radiation was attributed to its high sensitivity to radiation [32] leading to follicular disruption, thrombosis and degeneration of the vascular system due to inflammation, partial epithelial regeneration and cellular fibrosis and necrosis [33, 34]. Meanwhile, it was found that treatment with MO Extract maintained the thyroid function by reversing the effect of Elt.+IR on the thyroid gland. In agreement with this, Kaplan and Dosiou [35] reported that the presence of the caffeic, rosemary, and chlorogenic acids in the aqueous extracts of *M. officinalis* regulated the action of iodothyronine deiodinase which is responsible for the formation and degradation of T3 and restoring the euthyroid status thus confirming its antithyroid activity. Additionally, Hameed et al. [36] showed that the thyro-suppressive effect of *M. officinalis* was attributed to the high concentration of rosmarinic acid which effectively blocks TSH binding to the receptor by acting on the hormones and the receptor itself.

Functionally, both thyroid hormones and the liver are important to each other. Metabolism of thyroid hormones occurs in the liver, where they are broken down into glucuronides and sulfides that are excreted in the bile) [37]. Moreover, the liver is a major site for the production of albumin, transthyretin and thyroxine-binding globulin proteins that bind thyroid hormones [38]. Consequently, a hyperthyroid state is associated with various chronic disorders such as cardiovascular diseases, diabetes and liver diseases [39], and impaired both the hepatic structure and function [4, 40]. Additionally, the prevalence of liver dysfunction in patients with hyperthyroidism has been estimated to be between 37% and 77.9% [41].

In harmony with previous studies [42, 43], the current results elicited that thyrotoxicosis augmented the activities of both ALT and AST along with a sharp decrease in the levels of total protein, albumin and globulin. This was confirmed by the damaged structural integrity of the liver manifested by hepatocyte degeneration and coagulative necrosis. The hepatic dysfunction associated with hyperthyroidism was attributed to improper exposure to high levels of thyroid hormones, hepatocyte anoxia and apoptosis resulting from excessive energy demand associated with hypermetabolic state provoking mitochondrial generation of the reactive oxygen species (ROS) as well as mitochondrial apoptotic pathway, hepatocytes degeneration subsequent to degradation of glycogen and protein in addition to hepatic necrosis following thyrotoxicosis cardiac failure [44, 45]. On the contrary, treating the hyperthyroid rats with MO Extract remarkably alleviated the hepatic structure and dysfunction appeared clearly by the significant reduction in the activities of the serum transaminases (AST and ALT) coupled with enhancement of the levels of the hepatic proteins. Kim et al. [46] indicated that *M. officinalis (lemon Balm)* effectively attenuated liver damage in a model of Nonalcoholic Steatohepatitis assuring its hepatoprotective potential. Additionally, it was found that rosmarinic acid, one of the active components of *M. officinalis*, ameliorated multi-organ dysfunction markers (liver) and decreased the transaminases (AST and ALT) activities in the serum of liver ischemia-reperfusion (I/R) model [47].

The hypermetabolic state together with the dysfunctional mitochondrial respiration correlated to hyperthyroidism disrupting tissues and cellular redox homeostasis which is the dynamic balance between the production of the prooxidant (ROS and reactive nitrogen species) and their elimination by the antioxidant defence system [48, 49]. Furthermore, their excessive accumulation coupled with insufficient antioxidant defensive mechanisms [50] is associated with oxidative stress and damage of the cellular biomolecules (lipids, proteins, and nucleic acids) concomitant with cellular and tissue dysfunction [51]. The obtained data exhibited a notable elevation in the levels of the oxidative stress markers MDA and NO with marked depletion of both non-enzymatic (GSH) and enzymatic (SOD, CAT, and GPx) antioxidants in the hepatic tissues of the hyperthyroid rats due to the prolonged ROS production as well as consumption of the antioxidant defence system components. These results are in line with previous results of Panda et al. [29] and Ashry et al. [52]. Additionally, Kochman et al. [53] reported that the oxidative damage of cellular membrane lipids in hyperthyroid was higher than in euthyroid individuals. Conversely, it was found that M. officinalis protected the endogenous antioxidant system and minimized lipid peroxidation owing to its antioxidant potential [54, 55]. Furthermore, Ghazizadeh et al. [9] and Draginic et al. [56] attributed the powerful antioxidant effect of the MO Extract to the higher content of phenolic compounds rosmarinic acid (RA), caffeic acid, cinnamic acid, gallic acid, and ferulic acid) besides, different flavonoids (luteolin, quercetin and others). These phytochemical components improve the scavenging of NO and free radicals together with hindering free radical chain reactions that propagate oxidative stress status, electron-donation ability, and chelation activity [57].

Owing to the outstanding role of Nrf2/Keap-1 as one of the major adaptive stress response pathways that maintained cellular homeostasis and initiated a defence mechanism against oxidative stress damage as well as its critical protective role in the liver during inflammatory, fibrogenic, and carcinogenic processes it was recognized as an important target for treating liver diseases [58, 59]. Respectively, parallel to several previous studies, it was reported that hyperthyroidism impaired the Nrf2/Keap-1 pathway [49, 60] leading to a time-dependent elevation in the total mRNA expression of Nrf2 in the liver and its subsequent downstream antioxidant (GSH) as an early cytoprotective response to attenuate the oxidative stress damage and restore redox homeostasis [61]. Additionally, the continuous production of ROS either from hyperthyroidism or from metabolism and drug detoxification in the liver itself triggered a sustained state of oxidative stress along with the exhaustion of the antioxidant system resulting in the activation of the Keap1 thus excessive upregulation in the mRNA expression of Keap1. Romanque et al. [61] explained that the downregulation of Nrf2 after activation and upregulation of Keap1 was attributed to the activation of Keap1 through its hypomethylation. Moreover, Somade et al. [62] reported a significant overexpression of both Nrf2 and Keap1 total mRNA in the hepatic tissues of rats intoxicated with MECE and attributed this to the cellular response against the MECE-induced oxidative stress accompanied with depletion of the antioxidant contents during this cytoprotective response.

The impaired Nrf2/Keap1 synergized with redox imbalance and promoted hepatic injury, inflammation and fibrosis [63]. The excessive hepatocytes damage activated hepatic macrophages and aggravate the release of pro-inflammatory mediators (cytokines, chemokines and growth factors) which in turn boosts the inflammatory response and progression of fibrosis [64]. Lyu et al. [65] revealed overexpression of hepatic pro-inflammatory genes (IL-6 and TNF-α) due to hepatic deficiency of Nrf2. Owing to this, the excessive production of pro-inflammatory cytokines in the hepatic tissues of the hyperthyroid rats is assured by the upregulated mRNA expression of both TNF-α and MCP1 which is consistent with the results of Bahtiyar et al. [44] and Ashry et al. [52]. Queck et al. [66] reported that activation of the hepatic macrophage during liver injury was associated with upregulation of the hepatic MCP-1 which in turn activates fibrogenic hepatic stellate cells (HSCs) [67]. Additionally, the pro-fibrogenic cytokine TGF-β produced by kupffer cells as well as the activated HSCs themselves promotes the proliferation and differentiation of HSCs into collagen-producing myofibroblasts augmenting the expression of α-SMA and eventually hepatic fibrosis [68, 69]. Similarly, the expression of the SDF-1 was upregulated in response to the hepatic injury leading to the production of α-SMA and collagen after activating the HSCs [70]. Moreover, the upregulated expression of Keap1 together with deactivation or deficiency of Nrf2 aggravates hepatic fibrosis via ROS and activation of HSCs [63, 71]. Parallel to these results, our results exhibited a pronounced upregulation in the mRNA expression of the pro and fibrogenic mediators including TGF-β1, SDF-1 and α-SMA in the hepatic tissues of the hyperthyroid rats.

Interestingly, it was found that MO Extract treatment alleviated the inflammatory response through suppression of the pro-inflammatory mediators and activated the Nrf2/Keap1 pathway which subsequently diminished the development and progression of fibrosis. These results are in line with several previous studies that proved the anti-inflammatory effect of *M. officinalis* through the suppression of pro-inflammatory markers [56, 72], scavenging the intracellular ROS and protecting cells from oxidative stress damage [73] in addition to the inhibition of enzymes implicated in the inflammatory process attributing this to their phytochemical components mainly rosmarinic acid [74]. Abd Allah et al. [75] demonstrated that *M. officinalis* extract activated Nrf2 in the hippocampus of epileptic rats. It is noteworthy that activation of the Nrf2 pathway enhanced the antioxidant defence system and minimize the production of ROS [46, 76]. Furthermore, Wardyn et al. [77] and Vasileva et al. [78] revealed that the activated Nrf2 hampered the inflammatory cascade not only by suppressing NF-κB and pro-inflammatory mediators but also by activating the anti-inflammatory cytokines. Additionally, it was found that Nrf2 activation alleviates hepatic fibrosis by modulating the antioxidant stress response and thus diminished DNA damage, hindering the inflammation triggered by the pro-fibrotic macrophage besides preventing activation of the HSCs by TGF-β1/Smad pathway confirming the anti-fibrotic potential of the Nrf2 [79].

## Conclusion

In conclusion, the obtained results exhibited that *Melissa officinalis* extract maintained thyroid functions and alleviated hyperthyroidism-induced hepatic damage by ameliorating its functional and structural integrity. Moreover, MO Extract enhanced the cellular redox homeostasis by attenuating the deranged redox balance, protecting the hepatic cells from oxidative damage along modulating the Nrf2/Keap-1 pathway. Additionally, MO Extract not only alleviated the inflammatory response through suppression of the pro-inflammatory mediators but also prevented hepatic fibrosis *via* Nrf2-mediated inhibition of the TGF-β1/Smad pathway. Collectively, these results may prove the hepatoprotective potential of MO Extract in a rat model of hyperthyroidism. The limitation of the present study is that we did not use a standard drug for hyperthyroidism. Additionally, we only measured the m-RNA expression without its relevant protein levels. However, further clinical experiments are needed to ensure the exact mechanism.

## Data Availability

All data obtained from this study are included in the current manuscript.

## Abbreviations

MO: *Melissa officinalis*
IR: Radiation
Nrf2: Nuclear factor erythroid 2-related factor 2
Keap-1: Kelch-like ECH-associated protein-1
TNF-α: Tumor necrosis factor-α
MCP-1: Monocyte chemoattractant protein-1
TGF-β1: Transforming growth factor-beta1
T4: Thyroxine
T3: Triiodothyronine
SOD: Superoxide dismutase
CAT: Catalase
GPx: Glutathione peroxidase
GSH: Glutathione
HO-1: Heme oxygenase
Gy: Gray
TSH: Thyroid stimulating hormone
NO: Nitric oxide
MDA: Malondialdehyde
TP: Total protein
Alb: Albumin
SDF-1: Stromal cell-derived factor-1
α-SMA: Alpha-smooth muscle actin
Elt: Eltroxin
ROS: Reactive oxygen species
